# Risk and Protective Factors Related to Mortality from Pneumonia among Middle-aged and Elderly Community Residents: The JACC Study

**DOI:** 10.2188/jea.17.194

**Published:** 2007-12-19

**Authors:** Yusuke Inoue, Akio Koizumi, Yasuhiko Wada, Hiroyasu Iso, Yoshiyuki Watanabe, Chigusa Date, Akio Yamamoto, Shogo Kikuchi, Yutaka Inaba, Hideaki Toyoshima, Akiko Tamakoshi

**Affiliations:** 1 Department of Health and Environmental Sciences, Kyoto University Graduate School of Medicine.; 2 Department of Clinical Informatics, Kansai Rosai Hospital.; 3 Department of Public Health, Osaka University Graduate School of Medicine.; 4 Department of Epidemiology for Community Health and Medicine, Kyoto Prefectural University of Medicine.; 5 Department of Food Sciences and Nutrition, Nara Women's University Faculty of Human Life and Environment.; 6 Department of Infectious Disease Research Division, Hyogo Prefectural Institute of Public Health and Environmental Sciences.; 7 Department of Public Health, Aichi Medical University; 8 Department of Epidemiology and Environmental Health, Juntendo University of School of Medicine; 9 Department of Public Health/Health Information Dynamics, Nagoya University Graduate School of Medicine.; 10 Division of Clinical Trials, National Center for Geriatrics and Gerontology

**Keywords:** Pneumonia, Cohort Studies, Residence Characteristics, Blood Transfusion, Smoking

## Abstract

**BACKGROUND:**

There have been few systematic investigations into risk and protective factors for pneumonia related mortality for community residents. This study investigated these factors utilizing a large cohort study on Japanese community residents.

**METHODS:**

Subjects, 110,792 individuals (aged 40-79 years) enrolled in 1988-1990, were followed until death, or when they moved away from the surveyed communities, or the end of 2003. Pneumonia death was defined following 480-486 (International Classification of Diseases, 9th Revision) or J12-J18 (10th Revision). Age-adjusted and multivariate hazard ratios were calculated along with 95% confidence intervals using the Cox proportional hazards model.

**RESULTS:**

With 1,112,747 person-years of the study, a total of 1,246 persons died of pneumonia. We found history of blood transfusion (multivariate hazard ratio=2.0 [95% confidence interval: 1.7-2.4]) was a potent novel risk factor. Walking 0.5-1 hour/day (0.8 [0.6-1.0]), 1+ hour/day (0.7 [0.6-0.8]), and/or a history of pregnancy (0.6 [0.4-0.9]) were found to reduce pneumonia mortality. A large body mass index (BMI) (25+kg/m^2^) was a protective factor (0.7 [0.5-0.8]), while low BMI (<18) was confirmed as a risk one (2.1 [1.7-2.6]). Smoking was an important preventable risk factor (1.6 [1.3-1.9], population attributable risk proportion=14%), and its cessation reduced risk (0.7 [0.5-1.0]) to levels comparable to never-smokers (0.7 [0.5-1.0]).

**CONCLUSIONS:**

The risk and protective factors ascertained here for pneumonia mortality among community residents, history of blood transfusion, large BMI, and walking habits, warrant further study. Smoking cessation may effectively reduce pneumonia mortality.

Pneumonia poses a serious threat of mortality among the elderly. Aging is accompanied by a gradual physical decline along with alterations in many aspects of immune function. It is important for interventions to consider risk and protective factors in daily life that are associated with aggravating the prognosis of pneumonia. Although many studies on risk factors among hospitalized patients as well as case-control studies have been conducted,^1^^-^^5^ there have been few systematic investigations into community residents' risk factors: smoking,^6^^-^^8^ medical history such as heart disease, stroke, and diabetes mellitus,^7^^,^^8^ expiratory volume,^8^ and lower body mass index (BMI) ^7^^,^^9^ are some. These studies were relatively small population studies, and lifestyle influences were largely uncharted. Some lifestyle habits have been reported to be protective against physical decline (including immune response).^10^^-^^12^

The primary aim of the present study, therefore, was to identify factors determining susceptibility or resistance to pneumonia death among community residents. To achieve this, our study has taken advantage of the data available from a large cohort study.

## METHODS

### Study Cohort

The Japan Collaborative Cohort Study for Evaluation of Cancer Risk (JACC Study), sponsored by the Ministry of Education, Science, Sports and Culture of Japan, was established from 1988 through 1990 in 45 areas in Japan.^13^^, ^^14^ A total of 110,792 individuals (46,465 men and 64,327 women, aged 40-79 years) participated in municipal health screening examinations and completed a self-administered questionnaire.

### Questionnaire

A questionnaire form has the following items: age, measured weight and height, lifestyle (smoking, alcohol consumption, playing sports, walking) and medical history (stroke, hypertension, myocardial infarction, liver disease, renal disease, gallstone, diabetes mellitus, gastroduodenal ulcer, tuberculosis, cancer, blood transfusion, external injury requiring hospitalization, abdominal surgery, and pregnancy). From BMI, which were calculated using weight and height measurements, each participant was categorized as underweight (10<BMI<18), normal (18<BMI<23), mildly overweight (23<BMI<25), or overweight (25<BMI<33). Smoking and alcohol-consumption statuses were divided into three categories (current, past, never). Playing sports was categorized as <1, 1-2, 3-4, or >4 hours a week. Similarly, walking habit was categorized as <0.5, 0.5, 0.5-1, or >1 hour a day. Medical histories were inquired about, using a yes/no question as to whether the participant had a particular medical history. Those with non-marked or missing data in the questionnaire were not used in the analyses.

### Outcomes

Participants were followed up until death, or till they moved away from the surveyed community, or to the end of 2003. For mortality surveillance in each community, investigators systematically reviewed the death certificates of any participants, all of which were forwarded to the public health center in the area of residency. Mortality data were sent centrally to the Ministry of Health and Welfare and the underlying causes of death were coded for the National Vital Statistics according to the International Classification of Diseases, 9th Revision (ICD-9), from 1988 through 1994 and the International Classification of Diseases, 10th Revision (ICD-10), from 1995 through 2003, defining pneumonia deaths under 480-486 (ICD-9), and J12-J18 (ICD-10). The date of moving-out from the study area was also annually verified by the investigators in each area by reviewing population-register sheets of the cohort members. The present study was approved by the ethics committees of Nagoya University and the University of Tsukuba.

### Data Analysis

The annual mortality rates (AMR) for pneumonia as a national average were calculated from national statistics using the number of pneumonia deaths^15^ and the population in each age group.^16^ Age-adjusted and multivariate hazard ratios (HRs), along with 95% confidence intervals (CIs), were calculated using the Cox proportional hazards model. For serially-categorized variables (BMI, playing sports and walking), associations between pneumonia mortality and a linear trend for these variables were tested. In the multivariate analyses of medical histories and lifestyle factors, baseline age and history of diabetes mellitus were adjusted, and participants with a history of stroke were excluded from the analyses, because these factors are well confirmed risk factors for pneumonia death.^6^^,^^7^^,^^17^

Additionally, we calculated the population attributable risk proportion (PARP) to estimate preventable pneumonia. The PARP was calculated using HRs for each significant factor and the proportion of exposure population (Pe) as: PeX(HR - 1) / [PeX(HR - 1) + 1]. HRs were calculated from a multivariate analysis that contained all significant risk factors (baseline age, low-BMI, smoking, little walking, and history of stroke, diabetes mellitus, tuberculosis, cancer and blood transfusion) as variables.

In the final model, the relationship between smoking cessation and pneumonia mortality was examined by a multivariate analysis that contained all significant risk factors (baseline age, low-BMI, little walking, and history of stroke, diabetes mellitus, tuberculosis, cancer and blood transfusion), using current-smoker as the reference category.

## RESULTS

### Demographic Characteristics

Demographic characteristics of the cohort are provided in Table 1. During the observation period of 1,112,747 person-years, 16.0 % (10,367 men and 7,330 women) of the participants died, and 4.3 % (1,773 men and 2,992 women) were lost to follow-up because they moved out of the study areas. A total of 1,246 (791 men and 455 women) died of pneumonia. AMR for pneumonia increased with age, and AMRs in men were more than twice those in women for all age classes (Table 2). Generally, AMRs of the cohort were stably lower than those of the national average, suggesting a possibility that the present study failed to detect some of the pneumonia cases in the cohort. This is especially true for those who moved from their original residence to long-term hospitalization.

**Table 1.** Demographic data of the study cohort.

	Men	Women
No.	46465	64327
Observed person-years	455263	657484
Age (year, mean ± standard deviation)	57.6 ± 10.2	57.9 ± 10.2
Height (cm, mean±standard deviation)	162.8 ± 6.6	151.0 ± 6.0
Weight (kg, mean ± standard deviation)	60.3 ± 8.9	52.2 ± 7.9
Body mass index (kg/m^2^, mean ± standard deviation)	22.6 ± 3.3	22.6 ± 3.9

Body mass index (kg/m^2^)*		
10.0-17.9	1630 (3.7)	2628 (4.4)
18.0-22.9	23672 (53.9)	29958 (50.1)
23.0-24.9	10554 (24.0)	13848 (23.1)
25.0-32.9	8046 (18.3)	13400 (22.4)

Smoking*		
Current	22459 (52.0)	3102 (5.6)
Fomer	11681 (27.0)	969 (1.7)
Never	9061 (21.0)	51521 (92.7)

Alcohol consumption*		
Current	33278 (74.9)	14236 (24.5)
Fomer	2814 (6.3)	997 (1.7)
Never	8320 (18.7)	42979 (73.8)

Medical history*		
Stroke	914 (2.3)	585 (1.1)
Hypertension	8995 (21.7)	13568 (23.6)
Myocardial infarction	1312 (3.2)	1688 (3.0)
Liver disease	3078 (8.4)	3000 (5.9)
Renal disease	1606 (4.4)	2672 (5.3)
Gallstones	1675 (4.4)	3075 (5.8)
Diabetes mellitus	2880 (7.1)	2408 (4.4)
Gastroduodenal ulcer	9349 (23.0)	6398 (11.6)
Tuberculosis	2954 (7.7)	2745 (5.2)
Cancer	411 (1.1)	1051 (2.1)
Blood transfusion	3953 (10.3)	6031 (23.6)
External injury	10098 (29.0)	9472 (19.9)
Abdominal surgery	12298 (32.4)	12298 (28.3)
Pregnancy		45395 (96.5)

Exercise (playing sports)*		
<1 hour/week	25606 (68.6)	38939 (76.1)
1-2 hour/week	6279 (16.8)	7050 (13.8)
3-4 hour/week	2715 (7.3)	2785 (5.4)
More	2721 (7.3)	2361 (4.6)

Exercise (daily walking)*		
<0.5 hour/day	4456 (12.6)	5257 (10.8)
0.5 hour/day	6621 (18.7)	8598 (17.6)
>0.5-1 hour/day	6904 (19.5)	10031 (20.6)
More	17455 (49.3)	24883 (51.0)

*: No. (%)

**Table 2.** Annual mortality rates (AMR) for pneumonia in the study cohort, compared with the national average (Per 10,000 population).

Age (year)	Cohort							National Average
	
	No. of Participants	1990	2001	2002	2003	1990-2003		1999-2003
	
						Average (Range)		Average (Range)
						Men		
40-49	9829	0.8	-	-	-		0.1 (0.0-0.8)	0.4 (0.3-0.5)
50-59	13150	0.0	2.5	0.0	1.8		1.1 (0.0-3.1)	1.4 (1.2-1.7)
60-69	14907	2.0	3.5	0.9	0.9		3.5 (0.9-7.8)	6.4 (5.0-7.7)
70-79	7336	19.1	23.1	18.8	13.2		20.5 (11.0-34.0)	34.3 (26.2-45.0)
80-89	697	143.5	84.5	94.1	70.1		86.0 (17.8-143.5)	158.8 (134.4-184.8)
90+		-	153.5	176.1	131.9		158.8 (131.9-176.1)	463.4 (429.7-513.5)

						Women		
40-49	12835	0.0	-	-	-		0.2 (0.0-1.6)	0.2 (0.1-0.2)
50-59	19044	0.0	0.9	0.0	0.0		0.4 (0.0-0.9)	0.5 (0.4-0.6)
60-69	20587	2.4	1.2	1.2	1.2		1.1 (0.0-2.4)	2.2 (1.6-2.9)
70-79	10329	5.8	4.2	4.6	8.1		5.7 (1.8-9.6)	13.1 (9.5-16.9)
80-89	1028	19.5	22.8	30.0	41.4		30.5 (12.8-44.0)	74.1 (59.1-85.0)
90+		-	85.7	85.3	45.0		68.9 (45.0-85.7)	255.1 (228.4-290.3)

### Detection of Potential Risk and Protective Factors by Age-adjusted Analyses

Potential risk and protective factors for pneumonia death from the age-adjusted analyses are shown in Table 3. BMI was associated with pneumonia mortality: Low BMI elevated the risk while the reverse was true for large BMI. Current smoking and ex-drinking habits showed significant risk associations. With regards to medical history; stroke, myocardial infarction, diabetes mellitus, tuberculosis, cancer, and blood transfusion were all found to be associated with mortality risk, while a history of pregnancy in women significantly reduced mortality risk. Exercising (playing sports and walking) showed protective trends, although walking less than 0.5 hour/day habits increased mortality risk.

**Table 3.** Age-adjusted analysis of potential factors for pneumonia deaths.

	Men			Women			Total		
		
	No. (P-Y)*	HR (95% CI)	p	No. (P-Y)*	HR (95% CI)	p	No. (P-Y)*	p
Body mass index (kg/m^2^)								
10.0-17.9	95 (811)	1.9 (1.6, 2.4)	<.001	64 (488)	2.3 (1.7, 3.1)	<.001	2.1 (1.7, 2.5)	<.001
18.0-22.9	446 (3976)	1.0 (Reference)		192 (1904)	1.0 (Reference)		1.0 (Reference)	
23.0-24.9	102 (938)	0.7 (0.5, 0.8)	<.001	65 (599)	0.9 (0.7, 1.2)	0.329	0.7 (0.6, 0.9)	<.001
25.0-32.9	65 (633)	0.6 (0.5, 0.8)	<.001	53 (552)	0.7 (0.5 , 1.0)	0.026	0.6 (0.5, 0.8)	<.001

Trend			<.001			<.001		<.001

Smoking								
Current	360 (3319)	1.5 (1.2, 1.8)	<.001	29 (273)	1.5 (1.0, 2.2)	0.030	1.5 (1.3, 1.8)	<.001
Fomer	235 (2004)	1.2 (0.9, 1.4)	0.207	13 (89)	1.1 (0.5, 2.0)	0.894	1.2 (1.0, 1.4)	0.118
Never	134 (1121)	1.0 (Reference)		349 (3281)	1.0 (Reference)		1.0 (Reference)	

Alcohol consumption								
Current	442 (4094)	0.8 (0.7, 1.0)	0.041	60 (628)	0.8 (0.6, 1.0)	0.084	0.8 (0.7, 1.0)	0.009
Fomer	96 (741)	1.4 (1.1, 1.7)	0.014	20 (177)	2.7 (1.7, 4.3)	<.001	1.6 (1.2, 1.9)	<.001
Never	194 (1665)	1.0 (Reference)		314 (2861)	1.0 (Reference)		1.0 (Reference)	

Medical history								
Stroke	47 (315)	1.3 (1.1, 1.5)	0.005	20(156)	3.1 (2.0, 4.8)	<.001	2.4 (1.8, 3.0)	<.001
Hypertension	234 (2073)	2.1 (1.6, 2.9)	<.001	126 (1137)	0.8 (0.7, 1.0)	0.062	1.1 (0.9, 1.2)	0.302
Myocardial infarction	61 (481)	1.7 (1.3, 2.2)	<.001	18 (142)	0.8 (0.5, 1.3)	0.366	1.3 (1.1, 1.7)	0.016
Liver diseases	54 (441)	1.3 (1.0, 1.7)	0.091	18 (169)	0.9 (0.6, 1.5)	0.736	1.2 (0.9, 1.5)	0.225
Renal diseases	30 (235)	1.2 (0.8, 1.7)	0.334	17 (180)	0.9 (0.6, 1.5)	0.713	1.1 (0.8, 1.5)	0.613
Gallstones	35 (275)	1.1 (0.7, 1.5)	0.792	22 (185)	0.8 (0.5, 1.2)	0.261	0.9 (0.7, 1.2)	0.579
Diabetes mellitus	74 (635)	1.4 (1.1, 1.8)	0.008	39 (330)	1.6 (1.2, 2.3)	0.004	1.5(1.2, 1.8)	<.001
Gastroduodenal ulcer	178 (1568)	1.1 (1.0, 1.4)	0.151	51 (451)	1.0 (0.7, 1.3)	0.882	1.1 (0.9, 1.3)	0.243
Tuberculosis	93 (798)	1.3 (1.0, 1.6)	0.039	37 (316)	1.5 (1.0, 2.1)	0.028	1.3 (1.1, 1.6)	0.004
Cancer	18 (153)	1.8 (1.1, 2.8)	0.019	17 (121)	1.9 (1.2, 3.1)	0.002	1.8 (1.3, 2.5)	0.001
Blood transfusion	125 (1005)	1.9 (1.6, 2.3)	<.001	63 (527)	1.8 (1.4, 2.4)	<.001	1.9 (1.6, 2.2)	<.001
External injury	174 (1471)	1.1 (0.9, 1.3)	0.486	66 (602)	1.0 (0.8, 1.3)	0.977	1.1 (0.9, 1.2)	0.570
Abdominal surgery	227 (1833)	1.2 (1.0, 1.4)	0.035	109 (1054)	0.8 (0.6, 1.0)	0.070	1.0 (0.9, 1.2)	0.606
Pregnancy				343 (3237)	0.6 (0.4, 0.9)	0.019		

Exercise (playing sports)								
<1 hour/week	368 (3033)	1.2 (1.0, 1.5)	0.107	211 (1946)	1.2 (0.9, 1.7)	0.288	1.2 (1.0, 1.5)	0.051
1-2 hour/week	91 (789)	1.0 (Reference)		35 (281)	1.0 (Reference)		1.0 (Reference)	
3-4 hour/week	50 (448)	0.9 (0.6, 1.2)	0.432	21 (199)	0.9 (0.6, 1.4)	0.573	0.9 (0.7, 1.2)	0.619
More	54 (482)	0.8 (0.6, 1.1)	0.124	22 (217)	0.9 (0.8, 1.2)	0.551	0.8 (0.6, 1.1)	0.225

Trend			<.001			0.353		0.004

Exercise (daily walking)								
<0.5 hour/day	85 (601)	1.6 (1.2, 2.1)	0.001	47 (355)	1.7 (1.2, 2.5)	0.006	1.6 (1.3, 2.1)	<.001
0.5 hour/day	107 (895)	1.0 (Reference)		60 (508)	1.0 (Reference)		1.0 (Reference)	
>0.5-1 hour/day	108 (856)	0.8 (0.6, 1.1)	0.194	54 (475)	0.8 (0.6, 1.2)	0.253	0.8 (0.7, 1.0)	0.081
More	226 (1981)	0.7 (0.6, 0.9)	0.008	109 (1079)	0.7 (0.5, 1.0)	0.024	0.7 (0.6, 0.9)	<.001

Trend			<.001			<.001		<.001

*: Number of participants and observed person-year (parentheses)

HR: hazard ratio

CI: confidence intervals

### Multivariate Analyses of Possible Risk and Protective Factors

In our multivariate analyses of medical histories and lifestyle factors (playing sports and walking), baseline age, sex, history of diabetes mellitus were adjusted, and participants with a history of stroke were excluded from the analyses. However, even with these adjustments most risk/protective factors identified by Table 3 remained unchanged (Table 4). Associations were also confirmed when early deaths within 5 years of follow-up were excluded (low BMI, multivariate hazard ratio=2.0 [95% confidence interval: 1.6-2.6]; smoking habit, 1.8 [1.4-2.3]; blood transfusion, 1.8 [1.4-2.3]; pregnancy, 0.6 [0.4-1.0]; walking 0.5-1 hour/day, 0.8 [0.6-1.0], and 1+ hour/day, 0.7 [0.6-0.9]). We also confirmed that a daily drinking habit, even a heavy (≥69.0 g/day) one (1.0, [0.8-1.3]), was not significantly associated with pneumonia mortality risk.

**Table 4.** Multivariate analysis* of possible factors for pneumonia deaths.

	Men		Women		Total	
		
	HR (95% CI)	p	HR (95% CI)	p	HR (95% CI)	p
Body mass index (kg/m^2^)						
10.0-17.9	2.0 (1.5, 2.6)	<.001	2.3 (1.6, 3.1)	<.001	2.1 (1.7, 2.6)	<.001
18.0-22.9	1.0 (Reference)		1.0 (Reference)		1.0 (Reference)	
23.0-24.9	0.6 (0.5, 0.8)	<.001	0.8 (0.6, 1.2)	0.290	0.7 (0.5, 0.8)	<.001
25.0-32.9	0.6 (0.5, 0.9)	0.003	0.7 (0.5, 0.9)	0.023	0.7 (0.5, 0.8)	<.001

Trend		<.001		<.001		<.001

Smoking						
Current	1.6 (1.2, 2.0)	<.001	1.4 (0.9, 2.2)	0.138	1.6 (1.3, 1.9)	<.001
Fomer	1.2 (0.9, 1.5)	0.167	1.4 (0.7, 2.7)	0.351	1.2 (1.0, 1.5)	0.109
Never	1.0 (Reference)		1.0 (Reference)		1.0 (Reference)	

Alcohol consumption						
Current	0.8 (0.7, 1.0)	0.058	0.9 (0.6, 1.2)	0.370	0.8 (0.7, 1.0)	0.043
Fomer	1.1 (0.8, 1.5)	0.695	2.8 (1.6, 4.7)	<.001	1.3 (1.0, 1.7)	0.099
Never	1.0 (Reference)		1.0 (Reference)		1.0 (Reference)	

Medical History						
Hypertension	1.3 (1.1, 1.5)	0.010	0.8 (0.6, 1.0)	0.054	1.1 (0.9, 1.2)	0.443
Myocardial infarction	1.7 (1.2, 2.4)	0.001	0.8 (0.5, 1.5)	0.522	1.3 (0.9, 1.8)	0.183
Tuberculosis	1.4 (1.1, 1.8)	0.012	1.7 (1.1, 2.4)	0.009	1.5 (1.2, 1.9)	<.001
Cancer	1.7 (1.0, 3.0)	0.050	2.6 (1.5, 4.3)	<.001	2.1 (1.4, 3.0)	<.001
Blood transfusion	2.1 (1.7, 2.7)	<.001	1.9 (1.4, 2.5)	<.001	2.0 (1.7, 2.4)	<.001
Pregnancy			0.6 (0.4, 0.9)	0.017		

Exercise (playing sports)						
<1 hour/week	1.1 (0.8, 1.4)	0.502	1.4 (0.9, 2.2)	0.108	1.2 (1.0, 1.5)	0.129
1-2 hour/week	1.0 (Reference)		1.0 (Reference)		1.0 (Reference)	
3-4 hour/week	0.9 (0.6, 1.3)	0.487	1.5 (0.8, 2.7)	0.214	1.0 (0.7, 1.4)	0.951
More	0.7 (0.5, 1.1)	0.122	1.2 (0.6, 2.2)	0.592	0.8 (0.6, 1.2)	0.283

Trend		0.003		0.381		0.002

Exercise (daily walking)						
<0.5 hour/day	1.1 (0.8, 1.6)	0.509	1.5 (1.0, 2.3)	0.082	1.3 (1.0, 1.7)	0.105
0.5 hour/day	1.0 (Reference)		1.0 (Reference)		1.0 (Reference)	
>0.5-1 hour/day	0.8 (0.6, 1.1)	0.273	0.7 (0.5, 1.1)	0.181	0.8 (0.6, 1.0)	0.096
More	0.7 (0.5, 0.9)	0.005	0.7 (0.5, 1.0)	0.051	0.7 (0.6, 0.8)	<.001
Trend		<.001		<.001		<.001

*: Adjusted for age and history of diabetes mellitus. Persons with history of stroke were excluded.

HR: hazard ratio

CI: confidence interval

### Population Attributable Risk Proportion (PARP)

Among factors of baseline age, smoking, low BMI, little walking, and history of stroke, diabetes mellitus, tuberculosis, cancer, and blood transfusion, smoking accounted for PARP at 14.0%. Other factors were as follows: little walking (9.7%), low BMI (8.6%), history of blood transfusion (7.1%), stroke (3.1%), and tuberculosis (1.7%). These factors in total accounted for 44.2%.

### Risk Reduction by Smoking Cessation

In the final model, we examined the association of smoking cessation with pneumonia mortality risk. We used current smokers as the reference category with adjustment of significant variables (baseline age, low BMI, little walking, and history of stroke, diabetes mellitus, tuberculosis, cancer, and blood transfusion).

Our results showed that ex-smokers significantly reduced the risk of pneumonia mortality (multivariate hazard ratio = 0.7 [95% confidence interval: 0.5-1.0]) to levels comparable to those in never-smokers (0.7 [0.5-1.0]). Even when early deaths within 5 years of follow-up were excluded, the significant associations between cessation of smoking and reduction of pneumonia mortality risk (0.7 [0.5-1.0]) were confirmed.

For ex-smokers, smoking cessation for longer than 5 years before the baseline entry significantly reduced the risk of pneumonia mortality (0.7 [0.5-1.0]) to levels comparable to those in never-smokers. However, we could not accurately test the association between smoking cessation for 0-1 year, or 2-5 years with pneumonia mortality risk, because of the small sample sizes (0-1 years, n=12; 2-5 years, n=33). Therefore, we could not determine an effective minimum cessation period that significantly reduced pneumonia mortality risk.

## DISCUSSION

In this large cohort study, we found risk and protective factors that were significantly associated with pneumonia: Blood transfusion history was found to be a newly recognized risk factor while history of pregnancy, large BMI, and daily walking habits were protective factors. We also confirmed that low BMI was a risk factor while cessation of smoking was a protective one which might significantly reduce pneumonia mortality risk. These findings were observed using data from a cohort study of community residents and thus provide useful measures for intervention in respect to fatal pneumonia in the elderly.

History of blood transfusion might be related with immunomodulation. Before 1990, blood transfusions in Japan were conducted without white blood cell filters, irradiation, or screening for any bacterial or viral infections.^18^ Evidence from a variety of sources suggests that microchimerism by allogeneic blood transfusion increases the incidence of immunomodulation, ^19^^-^^22^ and allogeneic donor leucocytes have been considered one of the causes of the immunomodulation effect of transfusion.^23^ Such modulation, combined with aging, might result in an increased susceptibility to fatal conditions. It should be considered, however, that the experience of pregnancy, which is known to induce microchimerisms from fetuses,^24^ was a protective factor. This conflicting observation, also reported elsewhere,^25^ suggested that similar antigen exposure induces either a protective or detrimental effect to the host depending on the milieu of the immune system. Although the Pneumonia Severity Index (PSI) does not account for immunosuppression, pneumonia patients with immunosuppression such as hematological malignancies or bone marrow transplantation were reported to have significantly greater mortality.^26^

On the other hand, blood transfusion also could be considered as a surrogate marker for a variety of underlying factors that added to the pneumonia mortality risk. These underlying factors might be cofounders of the association between transfusion and pneumonia mortality risk, being associated with both the need for transfusion and the pneumonia mortality risk. Although we excluded the effect of medical histories surveyed in the questionnaire, there might be unidentified medical histories that engendered a spurious association between transfusion and pneumonia mortality risk. The persistent immunological influences of allogeneic blood transfusions before 1990 and its association with pneumonia mortality warrant further investigation.

BMI and some lifestyle factors were also found to be significantly associated with pneumonia mortality risk. Low BMI has been associated with increased risk of infection, possibly due to malnutrition or underlying illness,^7^^,^^9^ although obesity has also been suggested to elevate the risk associated with impaired T-and/or B-cell function.^27^ A cohort study in the United States reported a U-shaped relationship between BMI and immune dysfunction, showing that not only a low BMI but also a BMI with 27.0+ raise the pneumonia infection risk for men aged 40 to 75 years (compared with 21.0-22.9).^27^ Our data on Japanese did not support such a trend, although the range of our data on BMI was limited to between 10 and 33 in the cohort of community residents surveyed here.

We also found novel factors related to lifestyle: Exercise habits might be understood as an intermediate variable of being healthy, contrary to previous findings. Exercise habits were reported to reduce a wide range of mortalities,^28^ and some reports suggest that physical activity is associated with natural killer cell activity.^29^ These factors might also lead to functional independence in daily life. Adults aged 60 years and older who were functionally independent before admission were reported to be more likely to present with less severe pneumonia symptoms than patients who were functionally dependent before admission.^30^ Rather more systematic and detailed surveys will be needed to better elucidate the effects of these aspects of lifestyle.

The effects of alcohol consumption are also equivocal: one study reports chronic alcoholism increases pneumonia mortality risk,^31^ while a significant relationship between drinking habits and pneumonia infection was excluded in another.^27^ Our results from multivariate analyses showed ex-drinkers were associated with increased pneumonia mortality risk, while current-drinkers and even heavy-drinkers were not significantly associated with mortality risk. This might mean that a behavioral problem of a predisposition to alcohol abuse or other unspecified conditions that required quitting a drinking habit might be an important factor, as suggested in another study.^32^

Although we could not find an effective minimum smoking cessation period, we did demonstrate that smoking cessation might be effective in reducing, to a certain degree, the risk of pneumonia mortality. While the preventable portion of pneumonia infection by smoking cessation in the general population aged 18 to 64 years is reported to be 51%,^33^ our results for mortality showed that smoking cessation was still a significant measure (14%) against fatal conditions.

We confirmed that various medical histories are associated with pneumonia mortality risk,^6^^,^^7^^,^^17^ Additionally, our results newly revealed that some medical histories were associated with pneumonia mortality risk. Further etiological research will be needed on these newly identified medical histories.

The present study has several limitations. First, we did not detect any incident diseases or smoking status following baseline entry. Therefore any smoking cessation during follow-up was not included. Second, we could not take into account more detailed classifications of pneumonia death, potential confoundings from vaccinations, or other unmeasured items that might affect smoking cessation or pulmonary disease histories.^7^^,^^8^ For example, compared with current smokers, ex-smokers may be more health conscious, while some of them quit smoking because of illness; the net effect of these factors is uncertain.^34^ Furthermore, we could not confirm the reason for the blood transfusion, their frequency, the number of transfused blood units, or if there were unknown infectious agents. These factors might contain some unrecognized confounding effects related to the associations we found. Finally, there is a possibility that some of the pneumonia cases had been lost, specifically those who left their original communities to undergo long-term hospitalization.

In conclusion, the present study demonstrated possible risk and protective factors for pneumonia death based on a prospective mega-cohort of community residents. Our results showed that a history of blood transfusion significantly increased pneumonia mortality risk, and that low BMI and smoking habit were confirmed as risk factors. Contrarily, smoking cessation, walking habit, pregnancy in women, and a large BMI were found to reduce pneumonia mortality risk. Confirmation of the associations between the risk/protective factors suggested here and pneumonia mortality in other populations, and investigations related to their operative mechanisms await further study.
